# Distinguishable Prognostic miRNA Signatures of Head and Neck Squamous Cell Cancer With or Without HPV Infection

**DOI:** 10.3389/fonc.2020.614487

**Published:** 2021-02-10

**Authors:** Xiao-jie Luo, Min Zheng, Ming-xin Cao, Wei-long Zhang, Mei-chang Huang, Li Dai, Ya-ling Tang, Xin-hua Liang

**Affiliations:** ^1^ State Key Laboratory of Oral Diseases and National Clinical Research Center for Oral Diseases, West China Hospital of Stomatology, Sichuan University, Chengdu, China; ^2^ Department of Stomatology, Zhoushan Hospital, Wenzhou Medical University, Zhoushan, China

**Keywords:** HPV – human papillomavirus, HNSCC (head and neck squamous cell carcinoma), miRNA - microRNA, prognostic model, immune

## Abstract

Since their discovery in the 1990’s, microRNAs (miRNA) have opened up new vistas in the field of cancer biology and are found to have fundamental roles in tumorigenesis and progression. As head and neck squamous cell carcinoma (HNSCC) with positive human papillomavirus (HPV+) is significantly distinct from its HPV negative (HPV−) counterpart in terms of both molecular mechanisms and clinical prognosis, the current study aimed to separately develop miRNA signatures for HPV+ and HPV− HNSCC as well as to explore the potential functions. Both signatures were reliable for the prediction of prognosis in their respective groups. Then Enrichment analysis was performed to predict the potential biological functions of the signatures. Importantly, combining previous studies and our results, we speculated that HPV+ HNSCC patients with low signature score had better immunity against the tumors and enhanced the sensitivity of therapies leading to improved prognosis, while HPV− HNSCC patients with high signature score acquired resistance to therapeutic approaches as well as dysregulation of cell metabolism leading to poor prognosis. Hence, we believe that the identified signatures respectively for HPV+ and HPV− HNSCC, are of great significance in accessing patient outcomes as well as uncovering new biomarkers and therapeutic targets, which are worth further investigation through molecular biology experiments.

## Introduction

Based on the latest GLOBOCAN data (September 2018), head and neck squamous cell carcinomas (HNSCC), further classified as oral cavity, oropharynx, nasopharynx, larynx, and hypopharynx according to their primary site of origin, had the top eight combined incidence rate and was the 5th leading cancer by combined 5-year prevalence worldwide (1, 2). Despite the application of multimodal therapy and the marked improvement in overall survival (OS) for other tumors, the 5-year OS rate of HNSCC patients with locally advanced disease remains poor (25–40%) (3). Apart from excessive alcohol drinking and cigarette smoking, human papillomavirus (HPV) infection with increasing attention is also considered to be an important risk factor in HNSCC, particularly oropharyngeal tumors (4). In western world, alcohol and tobacco induced HNSCC is decreasing, while the incidence of HPV associated HNSCC, especially oropharyngeal, is significantly increasing in younger population (5). Accumulating evidence has showed that HPV positive (HPV+) HNSCC is significantly dissimilar to its HPV negative (HPV−) counterpart at the molecular level, with the respects of genetic, epigenetic, and protein expression profile (6–8). Furthermore, HPV+ is closely related with favorable overall survival (OS) in oropharyngeal squamous cell carcinoma (OPSCC), clinically. And as for non-OPSCC in HNSCC, the effect of HPV status on OS is controversial (9). However, the treatments for HPV+ and HPV− HNSCC patients remain almost the same, in spite of the vital differences between these two groups (10). Therefore, it is crucial to identify more specific and sensitive signatures for HPV+ and HPV− HNSCC respectively so as to develop better prognostic markers and therapeutic targets. It is indicated that HNSCC is more likely to be an epigenetic disease, rather than genetic (11), suggesting that miRNAs involved in epigenetic changes may be promising biomarkers in cancers (12–14).

MicroRNAs (miRNAs) are non-coding RNA molecules with about 22 nucleotides in length, which mainly act as negative regulators of target genes and have been shown to play an essential role in a range of biological functions, including cell proliferation, apoptosis, tumor growth, and metastasis (15). Nevertheless, to our knowledge, limited studies have been performed to identify the cancer specific miRNAs and tap the hidden value of their prognostic and therapeutic potentials, especially in HNSCC with division of HPV status.

**Figure 1:**
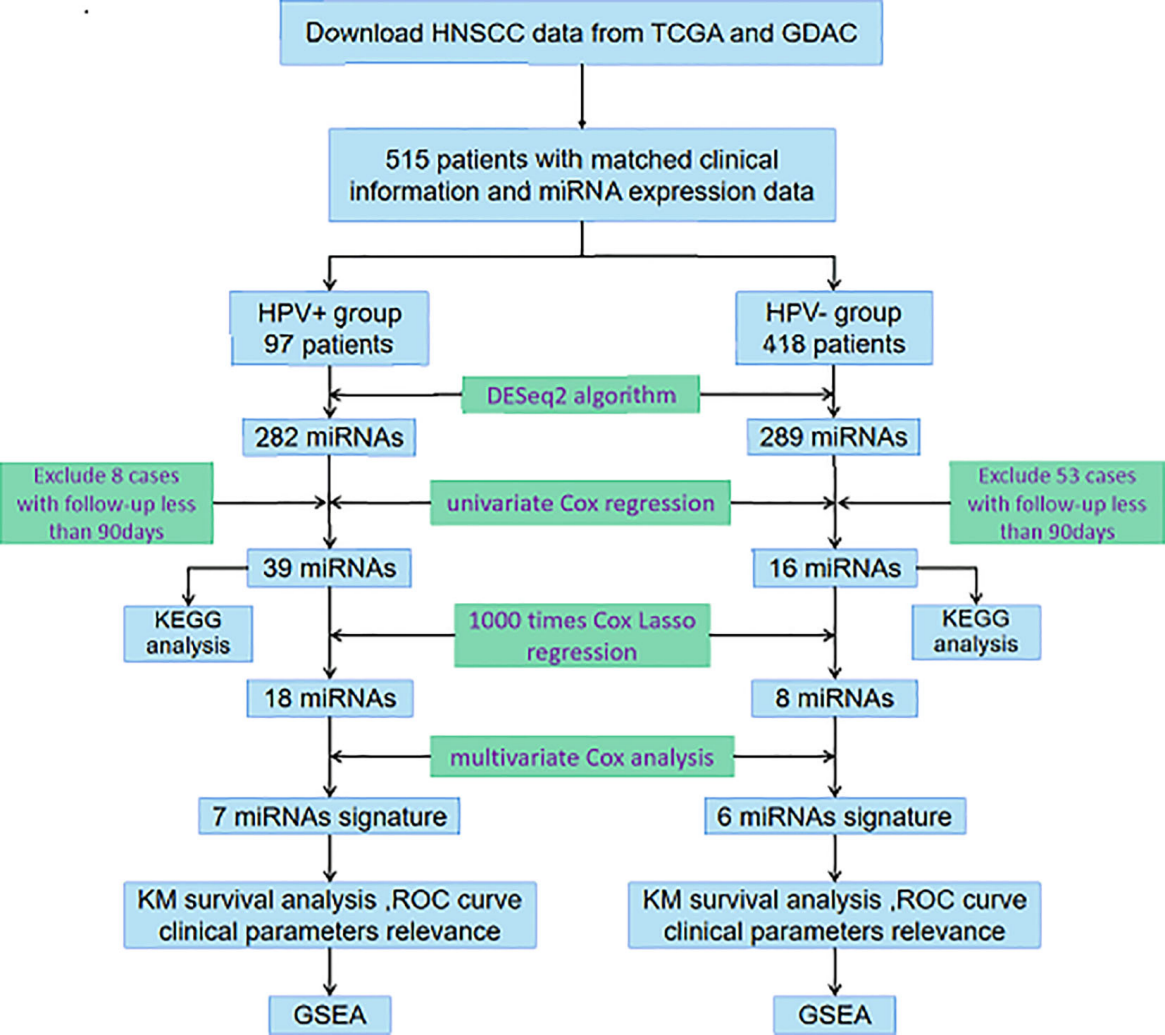
Flowchart illustrates the exploration procedure for the HNSCC prognostic miRNAs with division of HPV status.

In the current study, we integrated miRNA-Seq data and corresponding clinical follow-up information of HNSCC patients from The Cancer Genome Atlas (TCGA) database and Genome Data Analysis Centers (GDAC) server to identify features of miRNAs associated with prognosis. As it has been required to accurately discriminate between HPV-related and HPV-unrelated HNSCC, we then developed distinct miRNA signatures by dividing samples into HPV+ group and HPV− group. Enrichment analysis was further performed to investigate the potential functions, of which the results were truly distinguishable compared the two groups. The exploration procedure of the current study was illustrated in **Figure 1**.

## Materials and Methods

### Expression Data and Clinic Information Processing

The level three miRNA isoform expression quantification data and level three RNA-sequencing data (RNA-Seq-HTSeq-FPKM) of HNSCC patients was download from The Cancer Genome Atlas (TCGA; http://cancergenome.nih.gov/) on March 9, 2020. The matched patients’ clinical information was downloaded from the Broad Genome Data Analysis Centers Firehose server (https://gdac.broadinstitute.org/). The annotation of mRNAs was performed based on GENCODE (Release 33) and all miRNAs were renamed according to miRBase database v22 (www.mirbase.org) (16). The expression is taken as the median for the same gene or miRNA. All miRNAs with a row mean read less than or equal to one were ruled out. Then the raw counts of miRNA expression data were normalized by R (version 3.6.3)/Bioconductor (version 3.10) package “DESeq2” (17). The “DESeq2” and “apeglm” (18) was further used to detect the differentially expressed miRNAs between HPV+, HPV− and normal-adjacent control samples. The thresholds were set at the values of |log2 (fold change [FC])| >1 and p. adjust <0. 05. Deregulated miRNAs were compared with R package “VennDiagram”.

### Development of the miRNA-Based Prognostic Models

Cases with follow-up less than 90 days were excluded in advance and the prognostic value of each miRNA was calculated in the univariate Cox regression analysis using R package “survival” (p<0. 01). After primary filtration, Cox Least Absolute Shrinkage and Selection Operator (LASSO) regression with 10-fold cross-validation was built to pick out candidate miRNAs. Multivariate Cox regression was further performed using R package “survival” based on miRNAs disclosed in the above steps and constructed a prognostic risk score model. Risk score = *Expression (miRNAi) × Coefficient (miRNAi)*. Patients were divided into high risk and low risk groups according to the median risk score. Kaplan-Meier analysis was conducted using R package “survminer” and “survival.” The receiver operating characteristic (ROC) curve was obtained by R package “survivalROC.” Univariate and multiple Cox regression analysis were used to identify independent prognostic factors (p < 0. 05).

### Enrichment Analysis

Target genes of prognosis-related miRNAs were predicted by three databases (TargetScan, miRTarBase, miRDB) simultaneously. Kyoto Encyclopedia of Genes and Genomes (KEGG) pathway enrichment analysis for the target genes was conducted by R packages “org.Hs.eg.db” and “clusterProfiler” (19) (p < 0.05, q < 0.05). The matched mRNA expression data (FPKM) was divided into high-risk group and low-risk group by the mean risk score and was analyzed using GSEA version 4.0.3 (p < 0.05, FDR < 0.25).

## Results

### Patient Characteristics

A total of 515 HNSCC samples and 44 normal-adjacent control tissues with corresponding clinic data were included in this study. The cases were further grouped by HPV status and the basic characteristics of the patients were summarized in **Table S1** (in **Supplementary Materials**). The majority patients were male (73%), had no clinical metastasis (94.0%), with advanced stage disease (stage III or worse, 75.0%). Average age was 60.9 years (range 19–90) with a median of 61 years. Ninety-seven (18.8%) samples harbored HPV transcripts, among which 74 (76.3%) were HPV-16, and 23 (23.7%) were HPV-Other (16 HPV-33, 3 HPV-35, 3 HPV-18, and 1 HPV-56). Oropharyngeal tumors accounted for approximately 55.7% in the HPV positive samples compared with the oral cavity (33.0%), the larynx (6.2%), and the hypopharynx (5.2%). HPV+ patients were younger than HPV− patients (average of 57.8 *vs* 61.7 years); and the difference was statistically significant.

### Identification of Differentially Expressed miRNAs and Prognostic Signatures for HPV+ and HPV− Groups Respectively

**Figure 2:**
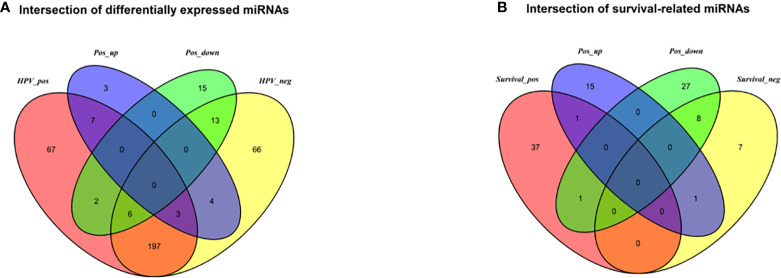
**(A)** Venn diagram of differentially deregulated miRNA sequences across groups; **(B)** Venn diagram of survival-related miRNAs. HPV_pos = differentially expressed miRNAs between HPV+ and normal-adjacent tissues; HPV_neg = differentially expressed miRNAs between HPV- and normal-adjacent tissues; Pos_up = up-regulated miRNAs compared HPV+ with HPV- tissues; Pos_down = down-regulated miRNAs compared HPV+ with HPV- tissues; Survival_pos = survival-related miRNAs in HPV_pos; Survival_neg = survival-related miRNAs in HPV_neg.

**Figure 3:**
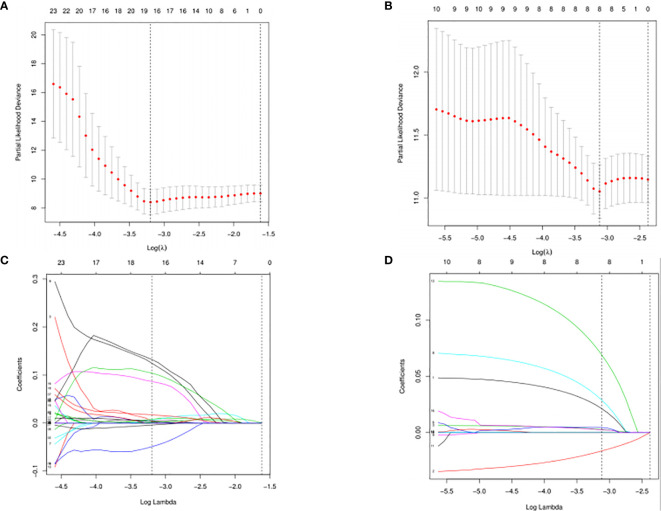
Cross-validated and lassopath of LASSO COX. HPV+ group: **(A)** and **(C)**; HPV- group: **(B)** and **(D)**. 18 variables were selected according to the lmin; log(lmin) = −3:20; **(C)** and **(D)** HPV- group, 8 variables were selected according to the lmin; log(lmin) = −3:12.

The DESeq2 algorithm identified differentially expressed miRNAs (p < 0.05) for three groups (HPV+ *vs* normal, HPV− *vs* normal, HPV+ *vs* HPV−), respectively. The lists of significantly deregulated miRNAs in each group were compared with Venn Diagram (**Figure 2A**). Given the significant distinctness between HPV+ and HPV− HNSCC at molecular level, we made our efforts to separately discover molecular biomarkers which could serve as available prognostic factors. Among the differentially expressed miRNAs, we further identified survival-related miRNAs (p < 0.01) for the two groups, accompanied by Venn Diagram (**Figure 2B**). Then the survival-related miRNAs were applied to 1,000 times Cox Lasso regression with 10-fold CV (**Figure 3**). Based on the screened-out miRNAs, multivariate Cox analysis developed distinct signatures for each group, respectively. No miRNAs were common between the two signatures. Risk score of each patient was calculated based on the expression and coefficient of miRNAs in the signatures. The formula of HPV+ group was as follows: Risk Score = (−0.00039788 × hsa-miR-378a-3p) + (−0.069642608 × hsa-miR-16-1-3p) + (−0.050112958 × hsa-miR-493-3p) + (0.238905909 × hsa-miR-380-5p) + (0.04184412 × hsa-miR-376c-3p) + (0.06043184 × hsa-miR-338-5p). The formula of HPV− group was as follows: Risk Score = (−0.035373051 × hsa-miR-135b-3p) + (0.144736959 × hsa-miR-605-5p) + (0.051610835 × hsa-miR-383-5p) + (0.011764307 × hsa-miR-518a-5p) + (0.006540076 × hsa-miR-1911-5p) + (0.074201442 × hsa-miR-548k). Negative values are bad for the occurrence of HNSCC, while positive values are favorable for it. The higher the risk score is, the worse the patient prognosis is.

### Evaluation of Clinical Outcomes for HPV+ and HPV− HNSCC Patients

**Figure 4:**
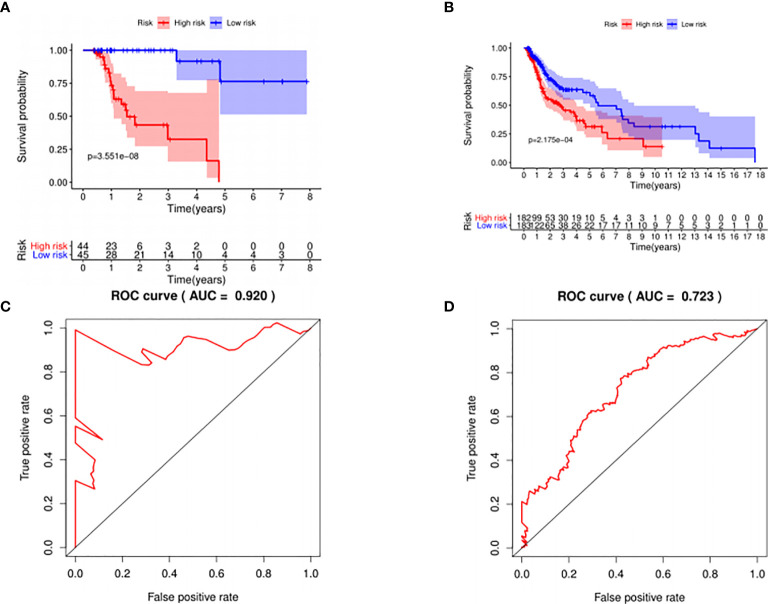
The prognostic value of the signature. HPV+ group: **(A)** and **(C)**; HPV- group: **(B)** and **(D)**. **(A, B)**: The overall survival curve of high risk score group and low risk score group. **(C, D)**: Receiver operating characteristic curve of the 5-year survival estimated via risk score.

**Figure 5:**
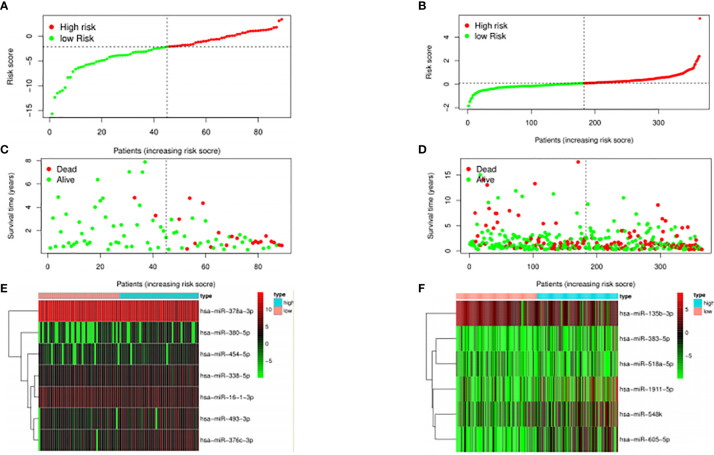
Expression profiles of the signature.

The median risk score was selected as a cutoff to further separate HPV+ and HPV− patients into high-risk and low-risk groups, respectively. As was shown in **Figures 4A, B**, both HPV+ and HPV− HNSCC patients in the low-risk group had longer OS (p < 0.001) than those of the high-risk group. And according to the 5-year survival receiving operating characteristic (ROC) curve, the area under curve (AUC) of risk score reached 0.920 in HPV+ group (**Figure 4C**) and 0.723 in HPV− group (**Figure 4D**). The detailed relationships between risk score, miRNAs expression, and survival information was shown in **Figure 5**.

Univariate and multiple regression analysis indicated both signatures for HPV+ and HPV− could serve as an independent predictor after other parameters were adjusted, including Tumor site, Age, Alcohol, Gender, Smoking, Grade, Clinical stage, Clinical T, Clinical N [**Table S2** (in **Supplementary Materials**)]. Relationships were also analyzed between the signatures and clinical characteristics [**Table S3** (in **Supplementary Materials**)]. Risk score of HPV+ was significantly lower (p < 0.001) in oropharynx than other sub-sites while there was no significant relationships between risk score of HPV− and clinical parameters.

### Functional Enrichment of the Survival-Related miRNAs

**Figure 6:**
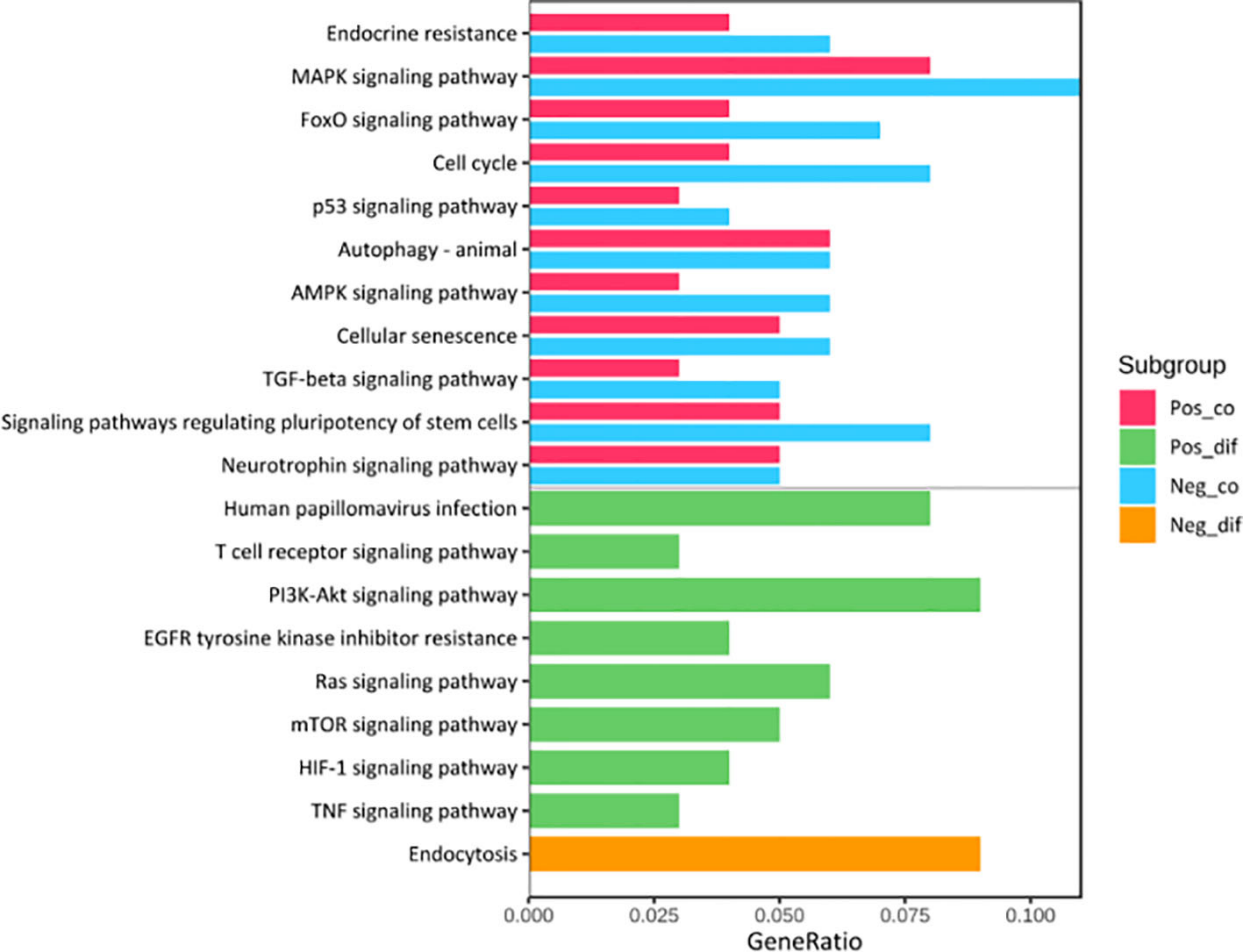
KEGG Pathway Enrichment Analysis. The pathways at the upper area divided by a grey line were the common ones between HPV+ and HPV- groups while those at the lower area were the distinct ones. Pos_co colored red stood for the enrichment outcomes of common pathways in HPV+ group and Neg_co colored blue stood for those in HPV- group. Pos_dif colored green were the enrichment outcomes of different pathways in HPV+ group and Neg_dif colored green were those in HPV- group.

**Figure 7:**
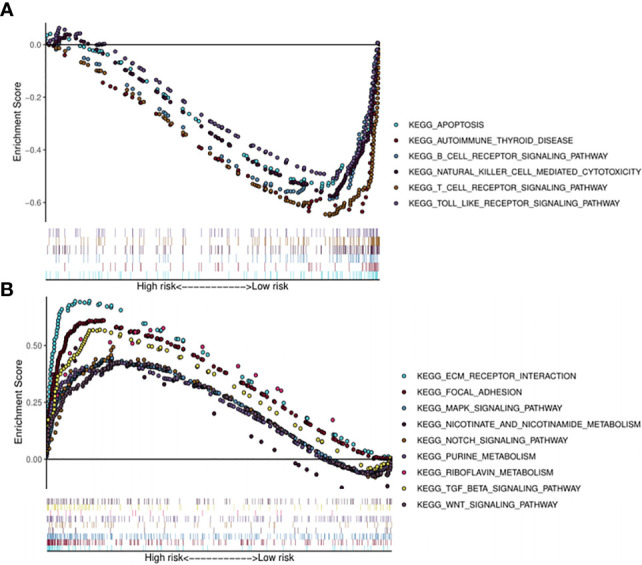
Risk score correlated enrichment gene analysis with multi- GSEA. HPV+ group: **(A)**; HPV- group: **(B)**.

Kyoto Encyclopedia of Genes (KEGG) pathway analysis was performed to assess the functional involvement of the survival-related miRNAs (**Figure 6**). Only the ones predicted by three databases (TargetScan, miRTarBase, miRDB) simultaneously were recognized as target genes for a given miRNA. As was shown in **Figure 6**, HPV+ group shared 11 common pathways with HPV− group, among which the gene ratio of HPV− group was generally higher than that of HPV+ group. And the selected different pathways in each group were also displayed at the lower area divided by the gray line. Furthermore, gene set enrichment analysis (GSEA) was implemented to investigate whether alternations of risk scores were linked to specific functional categories (**Figure 7**). GSEA of the signature score indicated that HPV+ samples with low risk score were mainly enriched in immune-related pathways, suggesting that HPV+ HNSCC patients with favorable prognosis might have better immune systems against the tumors (**Figure 7A**). As for HPV− group, high risk score appeared to be associated with metabolism and other vital oncogenic pathways showed in **Figure 7B**.

### Immune Cells Associated With Survival-Related miRNAs Signature for HPV+ and HPV− HNSCC Patients

Then, we further calculated the fractions of immune-related cells in the tumor micro-environment (TME) through CIBERSORTx and uncovered the relationships between the signature and these cells (**Table 1**). The results in HPV+ group showed that miRNAs associated with poor prognosis was negatively correlated with CD8+ T lymphocyte. Similarly, miRNAs associated with good prognosis was positively correlated with activated NK cells and T regulatory cells (Tregs). And the signature score was negatively correlated with T follicular helper cells (T_FH_) and T regulatory cells (Tregs).

**Table 1:** Relationships between signatures and immune cells.

HPV+ group				HPV− group			
miRNA	Immune cell	Cor	P	miRNA	Immune cell	Cor	P
hsa-miR-380-5p	T cells CD8	−0.489	0.002	hsa-miR-135b-3p	Macrophages M2	−0.224	0.001
hsa-miR-338-5p	T cells CD8	−0.454	0.004	hsa-miR-605-5p	T cells CD8	−0.189	0.007
hsa-miR-16-1-3p	NK cells activated	0.438	0.006	hsa-miR-605-5p	T cells CD4 memory activated	−0.277	<0.001
hsa-miR-378a-3p	T cells regulatory (Tregs)	0.662	<0.001	hsa-miR-605-5p	Macrophages M1	−0.182	0.009
Risk Score	T cells follicular helper	−0.51	0.001	Risk Score	T cells CD4 memory activated	−0.24	<0.001
Risk Score	T cells regulatory (Tregs)	−0.625	<0.001	Risk Score	T cells follicular helper	−0.187	0.007

For the signature of HPV− group, miR-605-5p related to unfavorable prognosis was found negatively associated with CD8+ T cells, macrophages M1, and activated memory CD4+ T cells, which acted as tumor suppressors. miR-135b-3p related to good prognosis was found negatively correlated with macrophages M2 which could promote tumor progression. And the signature score was negatively correlated with T_FH_ and activated memory CD4+ T cells. These findings implied that these miRNAs in the signature had potential for serving as biomarkers and therapy targets in HNSCC and deserved further investigation.

### Targeted mRNA of the Survival-Related miRNAs for HPV+ and HPV− HNSCC Patients

We have further found targeted mRNAs of survival-related miRNAs for HPV+ and HPV− HNSCC patients by TargetScan, miRTarBase, and miRDB. TCGA has been used to choose them and the negative correlation of miRNA and mRNA was applied to validate them. Then 1 mRNA in HPV− miRNA group and 10 mRNAs in HPV+ miRNA group were obtained and shown in **Table 2**.

**Table 2:** Targeted mRNA of the survival-related miRNAs for HPV+ and HPV− HNSCC patients.

	miRNA	mRNA	miRNALog_2_FC	mRNALog_2_FC
HPV+	miR-16-1-3p	CPS1	1.050639269	-1.010646906
	miR-16-1-3p	USP25	1.050639269	-0.718755746
	miR-16-1-3p	ITSN2	1.050639269	-0.684864346
	miR-16-1-3p	SLITRK4	1.050639269	-1.760387923
	miR-16-1-3p	DUSP5	1.050639269	-1.757895916
	miR-16-1-3p	WDR26	1.050639269	-0.781833209
	miR-338-5p	CSMD1	-2.24647131	1.859003192
	miR-338-5p	HOXA10	-2.24647131	3.622947307
	miR-376c-3p	BCL11A	-2.609241735	0.556674985
	miR-493-3p	XPR1	-1.197011744	0.620381708
HPV-	miR-605-5p	SLC16A1	-1.013638579	1.376851067

## Discussion

Over the past decade, the role of HPV has attracted increasing attention in HNSCC, and the significant differences of HPV+ HNSCC in prognosis and etiologic mechanisms from its HPV− counterpart have made it critical to describe and discuss the two subgroups separately whenever possible. Although this vital distinction mainly referred to oropharyngeal tumors within HNSCC, as this was the best-studied entity, HPV was also involved in 23.5% of oral cancer and 24% of laryngeal cancer cases (20). However, most of the identified prognostic signatures so far were still performed using total HNSCC samples regardless of HPV status (21–25). When HPV status was considered, most publications mainly focused on identifying differences in miRNA expression levels between HPV+ and HPV−, and HPV-associated oncogenic miRNA panel has potential utility in diagnosis and disease stratification of HNSCC (26–29). Therefore, to add the current knowledge, it is urgently required to discover robust prognostic factors for HPV+ and HPV− HNSCC which can provide new insights into finding potential prognosis biomarkers and therapeutic targets, respectively.

In the present study, we separately developed distinct miRNA signatures associated with OS of patients for the two groups. Both signatures were reliable for the prediction of prognosis in their respective groups according to the ROC curve. The lists of significantly deregulated miRNAs and survival-related miRNAs in each group were compared with Venn Diagram. Noteworthy were no survival-related miRNA in common compared HPV+ to HPV− groups, reinforcing their differences at the molecular level. Further, KEGG pathways analysis displayed that there were 11 common pathways between HPV+ group and HPV− group, and the gene ratio of HPV− group was generally higher than that of HPV+ group. In addition, GSEA of the signature score showed that HPV+ samples with low risk score were mainly enriched in immune-related pathways and HPV− group with high risk score appeared to be associated with metabolism and other vital oncogenic pathways. Previous studies have shown pronounced advances achieved in cancer immunotherapy. Despite the success, a relative lower overall clinical response of HNSCC was observed compared to other types of tumor treated with similar approaches, highlighting the need to gain better understanding of the complex immune landscape within TME of HNSCC (30, 31).

As our analysis implied that the identified signature of HPV+ group was highly correlated with immune-related pathways, we further calculated the fractions of immune-related cells in the TME and uncovered that survival-related miRNAs was correlated with CD8+ T lymphocyte, activated NK cells and Tregs and T_FH_. As components of the immune system, CD8+ T cells and NK cells have important roles in suppressing tumors by killing tumor cells with cytotoxic molecules, and the presence of tumor dense CD8+ T cells infiltration possibly represents pre-existing anti-tumor immune responses (32). T_FH_, a distinct subset of CD4+ cells, whose functions in cancers are rarely reported, seems to act as a protector in non-lymphoid tumors and hazardous factor in lymphoid tumors (33). Remarkably, the increased levels of Tregs, known to be capable of suppressing anti-tumor immunity, was observed in HPV+ HNSCC patients with better prognosis, opposite from the general findings in other tumor types. This was supported by previous publications. Lukesova et al. (34) found that higher infiltration of Tregs and lower ratio of CD8/Tregs associated with the better prognosis of HPV+ HNSCC. The reason of the different roles of Tregs were that Tregs may impair Th17-cell-dependent proinflammatory and also maintain HPV-positive status in HNSCC. Wansom et al. (35) showed that increased FoxP3+ Tregs infiltration closely associated with lower T stage and better survival in both HPV+ and HPV− HNSCC. The possible explanation is that pre-existing immunosurveillance against the HPV proteins actives the negative feedback of repressive mechanism, resulting in elevation of Tregs (3). We further identified a MEred module related with risk scores by WGCNA and performed GO and KEGG enrichment analysis for the module [**Figure S** (in **Supplementary Materials**)], whose results confirmed our findings that the miRNA signature of HPV+ was highly associated with immune systems. These provide a basis for the functional analysis in the immune-related pathways, indicating these miRNAs have crucial roles in immune infiltration within TME of HPV+ HNSCC, and the underlying mechanisms remain to be uncovered in the future.

Some identified miRNAs in HPV+ signature have been previously reported to be involved in progressions of several cancer types. A research showed that miR-378a-3p was related with favorable prognosis in ovarian cancer, which could suppress cell proliferation, promote cell apoptosis, and enhance the sensitivity to cisplatin by sponging MAPK1 and GRB2 (36). Wang et al. implied that miR-16-1-3p played an important role in gastric cancer by directly targeting Twist1 and suppressing Twist1-EMT pathways, and might also serve as available therapeutic targets for EMT process in other tumor types (37). miR-493-3p was found to induce apoptosis of ovarian cancer cells through targeting multiple genes, including AKT2, STK38L, HMGA2, ETS1, and E2F5 (38). It was reported that the inhibition of miR-380-5p could increase p53 expression and induce extensive cell apoptosis in human neuroblastoma cell lines (39). A study suggested that miR-376c-3p exerted an oncogenic role in hepatocellular carcinoma progression *via* repressing ARID2 (40). miR-338-5p appeared to induce invasion and metastasis of colorectal cancer partly through PIK3C3-related autophagy pathway and improved the proliferation and metastasis of malignant melanoma through targeting CD82 (41, 42). Herein, we hypothesized that the miRNAs might serve similar functions in HPV+ HNSCC, which deserved further investigation by molecular biology experiments.

Importantly, combining the outcomes of GSEA analysis for HPV− group and previous studies, it could be speculated that HPV− HNSCC patients with high risk score acquired resistance to therapies as well as dysregulation of cell metabolism responsible for poor prognosis. In order to satisfy the energy demands for malignant cell proliferation, tumors reprogram pathways of metabolism, which are recognized as hallmarks of cancers (43). Besides, it has been shown that cell adhesion to extracellular matrix (ECM) essentially linked to tumor cell resistance to radiation therapy, chemotherapy, as well as targeted drugs (44). Other vital oncogenic pathways were also enriched in those with high risk score, such as “MAPK SIGNALING PATHWAY,” “NOTCH SIGNALING PATHWAY,” “TGF BETA SIGNALING PATHWAY.” MAPK pathways essentially regulate malignant cell behavior in respect of cancer-related proliferation, invasion, migration, and survival, which are considered as viable targets for cancer therapy (45). Studies have revealed NOTCH pathway have a key role in HNSCC related to epithelial-mesenchymal transition (EMT) as well as immune system and its inhibition can decrease cell proliferation and migration, which are recognized as an attractive cancer therapeutic target (46). Apart from the ability of TGF-β pathway to impact diverse cellular processes including cell proliferation, invasion, and ECM remodeling, a research has shown TGF-β-mediated effects on squamous cell carcinoma are linked to metabolic reprogramming, which in particular play a vital role in the responses to anti-tumor therapeutic approaches (47). Thus, it can be inferred that HPV− HNSCC patients with high signature score may acquire more benefits from targeted medicine against the above pathways. The relationships between the signature of HPV− group and immune cells were also explored (**Table 1**). miR-605-5p and miR-135b-3p related to prognosis associated with CD8+ T cells, macrophages M1, and activated memory CD4+ T cells and macrophages M2. And the signature score was negatively correlated with T_FH_ and activated memory CD4+ T cells. These indicated that these miRNAs in the signature had potential to serve as therapy targets in HPV− HNSCC. However, as far as we know, there are limited studies towards the identified miRNAs in HPV-signature. A research implied that miR-605-5p promoted proliferation and invasion of non-small-cell lung cancer cell carcinoma *via* sponging TNFAIP3 (48). It was reported miR-135b-3p could inhibit cell clonogenicity and metastasis in triple-negative breast cancer by targeting RGMA (49). Nevertheless, in contrast to our findings, researches suggested that miR-383-5p acted as an inhibitor of cell proliferation and related with poor prognosis in several tumor types, such as ovarian, gastric, and breast cancer (50–52). Similarly, miR-518a-5p was reported to suppress proliferation and promote apoptosis of gastrointestinal stromal tumor while our results showed miR-518a-5p was correlated with poor prognosis of HPV− HNSCC patients (53).

Personalized treatment on the basis of HPV status with better efficacy and minimal side effects is on the horizon. Compared with previously established signatures irrespective of HPV status (21–25), the current study might be more accurate in predicting prognosis of HNSCC patients and, more importantly, better describe the potential molecular mechanisms by dividing samples into HPV+ group and HPV− group. According to the results, we speculated that HPV+ HNSCC patients with low signature score might have better immunity against the tumors and enhance the sensitivity of therapeutic interventions leading to improved prognosis, while HPV− HNSCC patients with high signature score acquired resistance to therapeutic approaches as well as dysregulation of cell metabolism responsible for poor prognosis. Thus, immunotherapy may be useful for HPV+ HNSCC patients with low signature score. We believe the identified signatures respectively for HPV+ and HPV− HNSCC, are of great significance in accessing patient prognosis as well as uncovering new biomarkers and therapeutic targets. However, specific limitations of our study should be mentioned. First, the present signatures based only on single public database, were not validated with an additional data set and the sample size of HPV+ group was relatively small. Second, despite the marked heterogeneity exists between HNSCC subtypes derived from different primary site, we did not separately construct type-specific signatures on account of limited sample size for a specific subtype. Finally, clinical specimen validation along with molecular biology experiments was not performed to uncover the potential mechanisms regulated by hub miRNAs which need to further investigate in the future.

## Data Availability Statement

The original contributions presented in the study are included in the article/**Supplementary Material**. Further inquiries can be directed to the corresponding authors.

## Author Contributions

X-JL, Y-LT, and X-HL conceived and designed the study. X-JL drafted the manuscript. X-JL analyzed and interpreted all the data. X-JL prepared the figures and tables. X-JL, M-XC, W-lZ, M-CH, LD, Y-LT, and X-HL reviewed and revised the manuscript. All authors contributed to the article and approved the submitted version.

## Funding

This work was supported by National Natural Science Foundation of China grants (Nos. 82073000 and 81972542), National Science Foundation of Sichuan Province (No. 2020JDRC0018 and 2020YFS0171), Clinical Project of West China College of Stomatoloy, Sichuan University (LCYJ2019-8), and Exploration and research projects of West China College of Stomatoloy, Sichuan University (LCYJ2020-YJ-1).

## Conflict of Interest

The authors declare that the research was conducted in the absence of any commercial or financial relationships that could be construed as a potential conflict of interest.
