# Supplementary material for: Distinguishable Prognostic miRNA Signatures of Head and Neck Squamous Cell Cancer With or Without HPV Infection
Source: Front Oncol. 2021 Feb 10;10:614487. doi: 10.3389/fonc.2020.614487 (PMC7902765; doi:10.3389/fonc.2020.614487)
Supplement: Supplementary file 1 [file DataSheet_1.pdf]

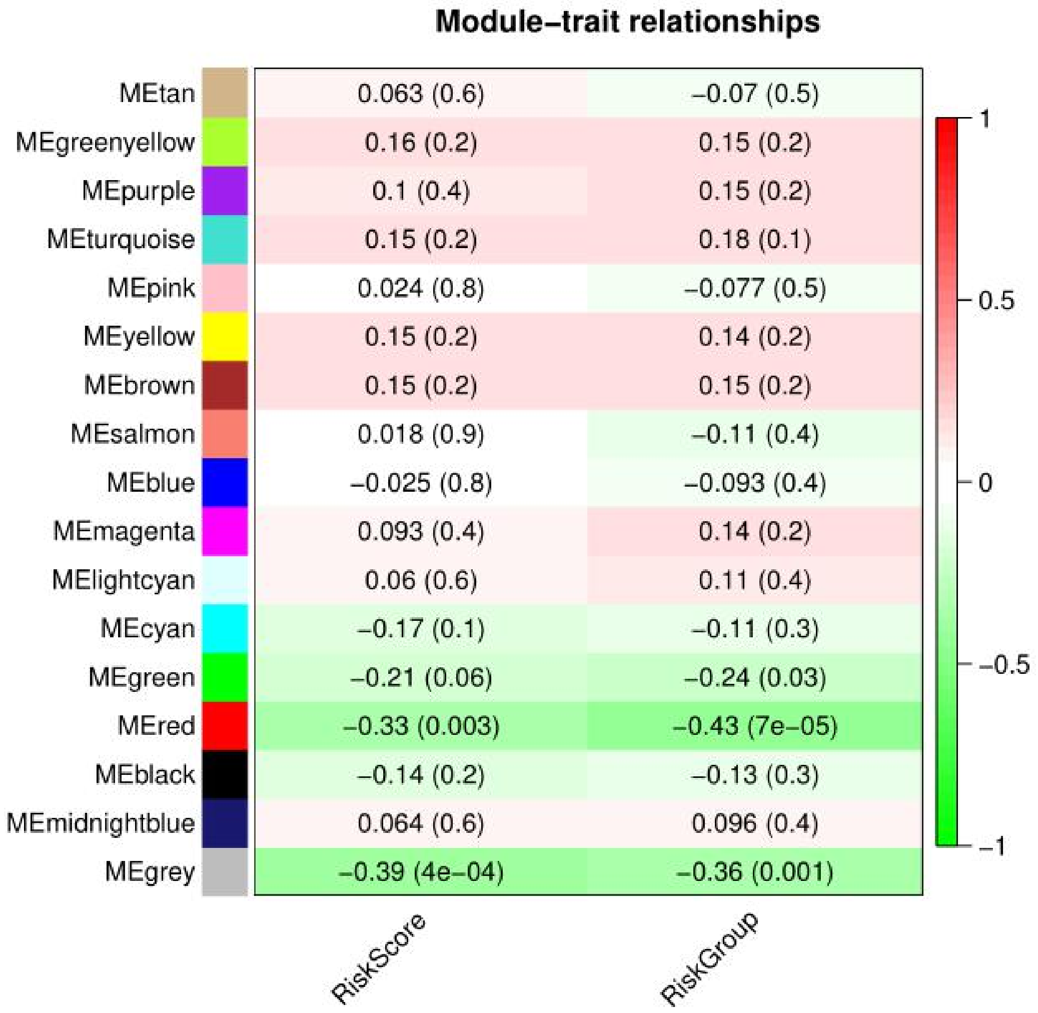

FigureS1 WCGCNA to identify modules related with risk score and risk group. Differentially expressed genes between HPV+ and normal-adjacent tissues were used to perform WCGNA.We identified MEred module significantly associated with both risk score and risk group for further enrichment analysis.

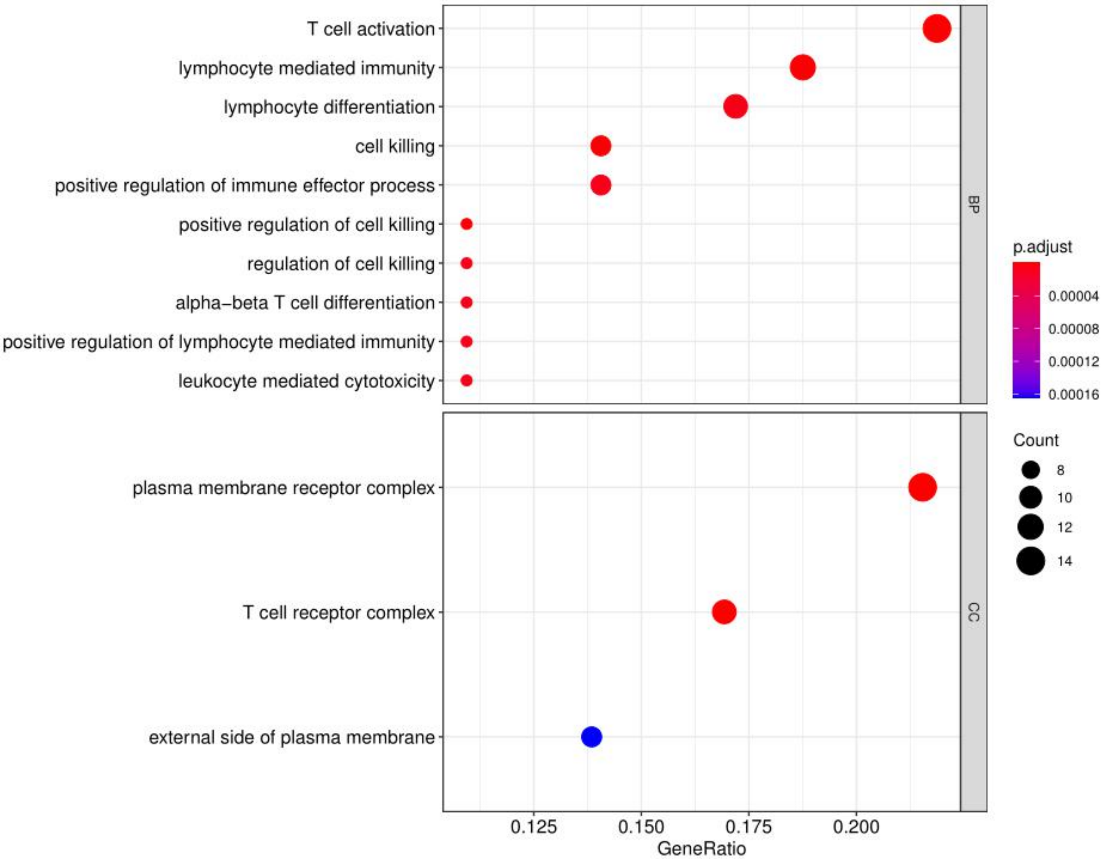

FigureS2 GO enrichment analysis of risk-related MEred module.

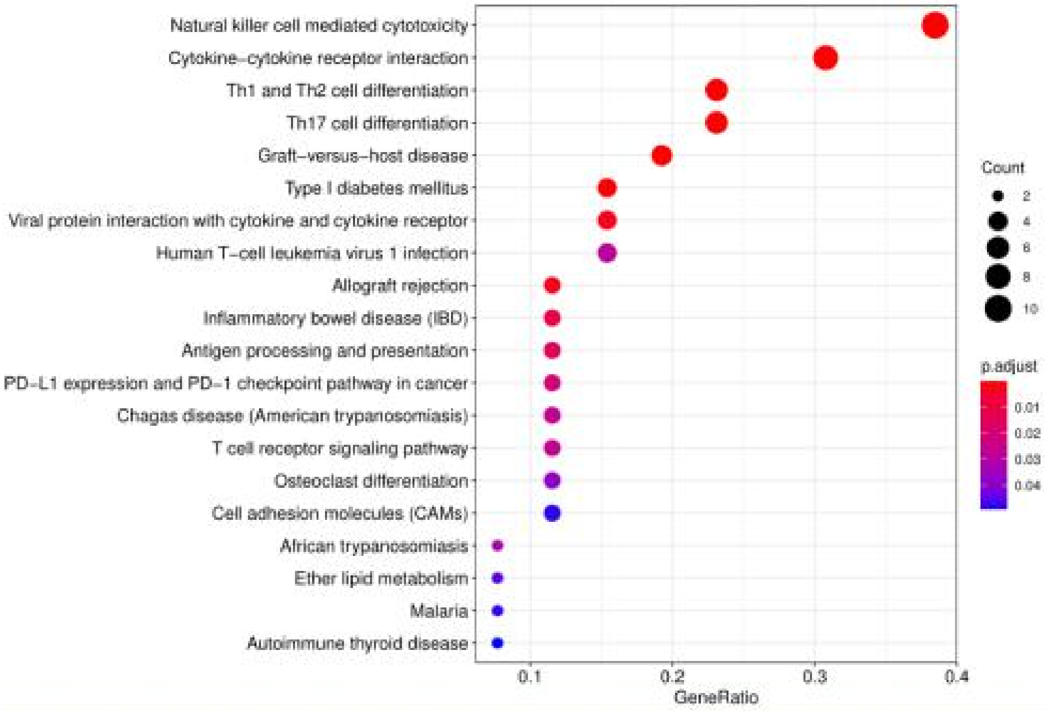

FigureS3 KEGG enrichment analysis of risk-related MEred module.
